# Uterine Artery Pseudoaneurysm after an Uncomplicated Vaginal Delivery: A Case Report

**DOI:** 10.3390/clinpract12050087

**Published:** 2022-10-12

**Authors:** Paul Böckenhoff, Patrick Kupczyk, Kira Lindner, Brigitte Strizek, Ulrich Gembruch

**Affiliations:** 1 Department of Obstetrics and Prenatal Medicine, University Hospital Bonn, 53127 Bonn, Germany; 2 Department of Diagnostic and Interventional Radiology, University Hospital Bonn, 53127 Bonn, Germany

**Keywords:** uterine artery pseudoaneurysm, postpartum hemorrhage, embolization

## Abstract

Uterine artery pseudoaneurysm (UAP) is a rare and potentially life-threatening vascular anomaly caused by inadequate sealing of a ruptured wall of a uterine artery. It mainly occurs after a traumatic lesion and can lead to delayed postpartum hemorrhage. We report a rare case of UAP after an uncomplicated vaginal delivery in a patient with a history of deep-infiltrating endometriosis. Selective coil embolization was successfully performed. UAPs should always be considered in cases of unexplained abdominal pain after surgery or childbirth with or without vaginal bleeding.

## 1. Introduction

Uterine artery pseudoaneurysm (UAP) is a rare and potentially life-threatening vascular anomaly caused by inadequate sealing of a ruptured wall of a uterine artery. The pain can vary from mild to acute abdominal. Thus, it may mimic and mask other entities, such as adnexal masses, septic miscarriage or endometritis. UAPs mainly occur after traumatic delivery, including caesarean section or surgical procedures such as hysterectomy, myomectomy, curettage or conization. It can be a rare cause of delayed postpartum hemorrhage, and occurs in approximately in 2–3/1000 births [1,2]. 

## 2. Case Presentation

A 30-year-old woman, gravida 1, para 1, was readmitted to our clinic because of increasing right lower abdominal pain that had started the previous day. She had an uncomplicated vaginal delivery 7 days ago with an estimated blood loss of 500 mL. Eighteen months ago, the patient had undergone laparoscopic endometriosis remediation for deep infiltrating endometriosis (rASRM IV; ENZIAN B3 FA) with adhesiolysis and excision of a right-sided ovarian cyst. During this operation, a chromopertubation was also performed due to a desire to have children. In the postoperative course, she initially had no pain.

**Figure 1:**
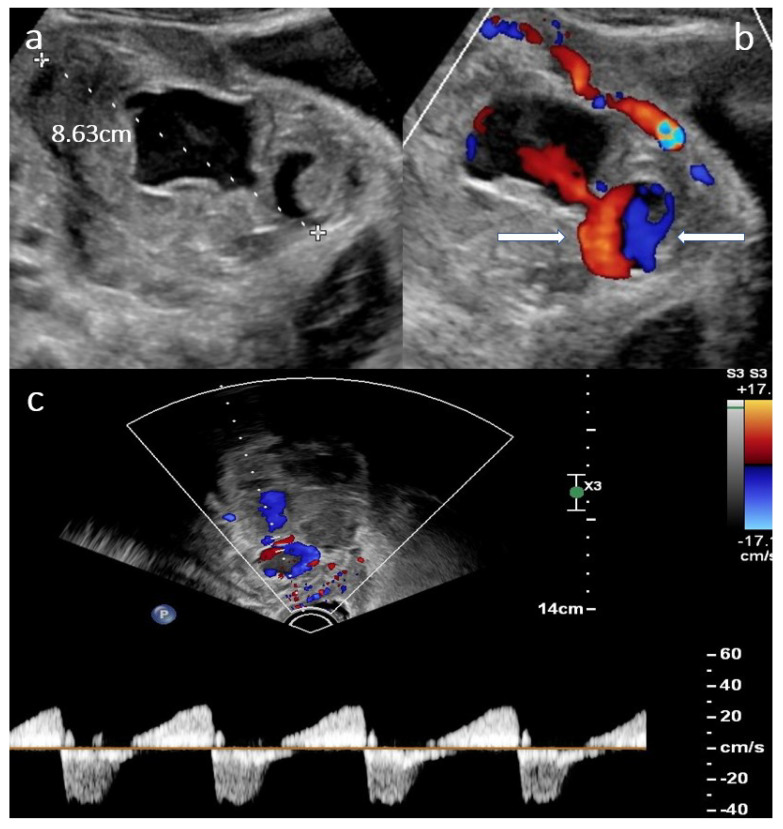
(**a**) Ultrasound findings in B-mode showing an anechoic lesion within an echogenic mass representing the UAP with surrounding hematoma. (**b**) Color Doppler showing Yin–Yang sign (between arrows). (**c**) Pulsed wave Doppler showing a to-and-fro pattern in the lower part of the image.

When presenting in our clinic, there were no signs of circulatory compromise (blood pressure 115/73 mmHg, pulse 72 bpm), no fever and no vaginal bleeding. Hemoglobin was 10.4 g/dL on the day of the new admission, and leucocyte levels were 7.3 G/l and 12.5 G/l, respectively. There was no free fluid in the abdominal cavity on transabdominal ultrasound. There was a pressure pain over the lower right abdomen with discrete guarding. An acute abdomen was ruled out. Upon transvaginal ultrasound, however, there was an echogenic mass of 8.6 cm in diameter on the right side, adjacent to the uterus, with a double-headed anechoic structure in the center, each cavity of approximately 3–4 cm in diameter. Color Doppler revealed a swirl of colors (Yin–Yang sign) and a pulse-synchronous blood flow from one cavity to the other as in arterial perfusion (Figure 1a–c).

**Figure 2:**
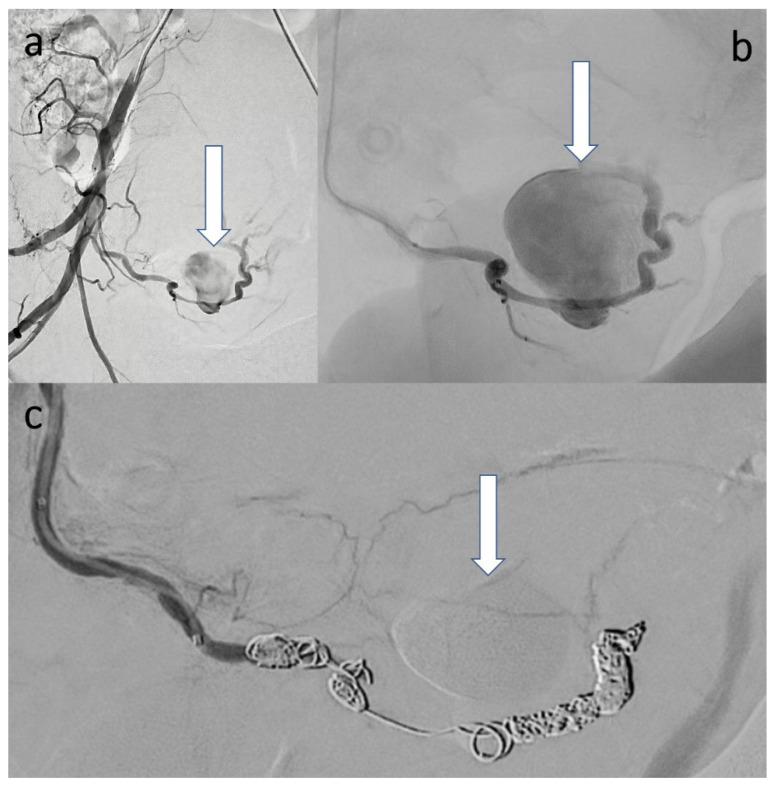
(**a**) Digital subtraction angiography (DSA) of the right hypogastric artery in 30° right anterior oblique projection demonstrating the large UAP (arrow). (**b**) Close-up of the UAP after superselective probing immediately before coiling (arrow). (**c**) DSA with total exclusion of the UAP after coil embolization (arrow).

Incidentally, the uterine cavity was slightly extended due to lochial stasis and the right ureter was mildly dilated. The CT scan revealed two hypodense, confluent lesions with complete contrast in the venous phase surrounded by a densely raised fringe with a total extension of about 9.4 × 6.8 cm corresponding to an intramural, double-lobed pseudoaneurysm with surrounding hematoma within the right lateral uterine wall. An angiographic intervention was performed. After cross-over probing of the right hypogastric artery, angiographic overview images were obtained, revealing the large, double-lobed pseudoaneurysm originating from the right uterine artery. The uterine artery was probed superselectively to the site of the findings, using a microcatheter system in a coaxial fashion. After unsuccessful embolization attempts with temporary and permanent particles (gelatin sponge and Embozene^®^ (900 μm)), the pseudoaneurysm was excluded from the circulation by coil embolization (6 microcoils in total) (Figure 2a–c).

**Figure 3:**
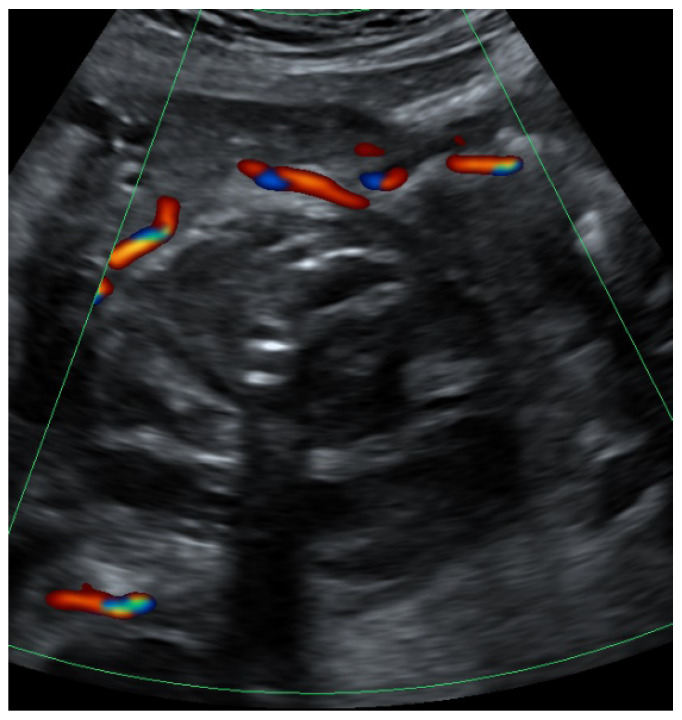
Color Doppler showing the findings 6 days after angiographic coil embolization with absence of intralesional flow, change in echogenicity and regreening of the hematoma.

Subsequently, neither further sources of bleeding nor collateralization of the pseudoaneurysm from the contralateral side could be detected. After short-term monitoring in the intensive care unit, the patient was transferred to the peripheral ward. Sonographic scans subsequently showed no blood flow in the area of the pseudoaneurysm. The day after the intervention, the patient reported pain in the right flank area and exhibited an elevated temperature up to 38.1 °C. Laboratory testing showed increased inflammatory parameters (C reactive protein 168 mg/dL, leukocytes 15.8 G/l). The right renal pelvis was still mildly dilated. Suspecting a right-sided pyelonephritis, a calculated antibiotic therapy was initiated. The further inpatient course was uneventful. After a total of 10 days, the patient was discharged from the hospital. Figure 3 shows the findings after intervention. Clinical and laboratory parameters are summarized in Table 1.

Four months later, a control MRI showed a regressive finding, now 5.2 cm in diameter, with no evidence of contrast and a clear diffusion restriction of the switched-off UAP. Even after 18 months, the patient is doing well. She has breastfed her child and has a normal menstrual period.

## 3. Discussion

UAP is a rare cause of delayed postpartum hemorrhage, it occurs in approximately in 2–3/1000 births [2]. In contrast to the mainly seeping bleeding after a caesarean section, a ruptured UAP usually results in acute, life-threatening bleeding, uterine, vaginal and/or intra-abdominal, after a seemingly uncomplicated initial postoperative period. Unlike a true aneurysm, a pseudoaneurysm does not have all three layers of the arterial wall. The differential diagnosis of a pseudoaneurysm includes acquired arteriovenous malformations (AVM), arteriovenous fistulas and direct vessel ruptures [3]. UAPs usually develop after iatrogenic damage to the vessel wall, after which there is an outflow of blood into the periarterial tissue, leading to the formation of a blood-filled cavity with surrounding hematoma communicating with the parent arterial lumen. The abovementioned Yin–Yang sign representing turbulent blood flow within the cavity as well as the to-and-fro pattern are sonographic signs suggestive of UAP [4,5].

UAPs can be difficult to detect, especially in cases without a history of operative trauma. There is a high risk of rupture; the exact time of this incident is not reliably predictable. Early detection is therefore mandatory, as rupture can lead to massive, potentially life-threatening intra-abdominal hemorrhage.

UAPs mainly occur after traumatic delivery including caesarean section [6,7] or other surgical procedures such as hysterectomy, myomectomy, curettage or conization [8,9,10]. However, UAPs may occur after uncomplicated vaginal deliveries and during pregnancy [11].

Usually UAPs can be detected by transabdominal and transvaginal ultrasound. Supplementary imaging, such as CT, can support the diagnosis and serve as a guidance for the interventional or surgical treatment strategy. In the past, most UPAs were treated by laparotomy or laparoscopy and arterial ligation. This was associated with surgical risks, including peritonitis, wound infection, postoperative adhesions, pain, scar discomfort, scar herniation and thrombosis [12]. Angiography with endovascular embolization has become the preferred treatment due to its high effectiveness and low risk at the same time. The overall complication rate for obstetric and gynecologic embolization procedures is 6–9%. Complications include hematoma at the groin puncture site, arterial dissection, and contrast-associated allergy or nephrotoxic effects. However, during pregnancy, MRI should be preferred due to radiation exposure [13,14].

In our case, the patient had undergone laparoscopy 18 months before the event due to infertility and an ovarian cyst. Deep infiltrating endometriosis was found during surgery. Whether endometriosis itself or the operation 18 months prior to delivery was a causing factor in the development of this UAP remains elusive. However, cases of UAP with endometriosis have already been described [15,16]. During pregnancy, endometriosis activity generally decreases and decidualization occurs. Neovascularity may develop in the decidualization reaction triggered by gravidity. It is possible that decidualization and subsequent neovascularity may have led to the development of a pseudoaneurysm. Moreover, there is a positive correlation between endometriosis and placenta praevia [17]. 

UAPs are potentially life-threatening, and must always be considered in cases of unexplained abdominal pain after surgery or after childbirth with or without vaginal bleeding.

## Figures and Tables

**Table 1:** Clinical and laboratory parameters.

	Before Birth	Day 7 after Birth (New Admission)	Day 8 after Birth (After Embolization)
C-reactive protein (CRP) (mg/dL)	/	/	168
leuocytes (G/l)	7.3	12.4	15.8
hemoglobin (G/l)	11.8	10.4	8.3
fever (°C)	no	no	38.1
vaginal bleeding	birth: 500 mL	no	no
systolic blood pressure (mmHg)	130	115	119
diastolic blood pressure (mmHg)	82	73	70
pulse (bpm)	79	72	70
drugs			Sultamicillin

## Data Availability

Not applicable.
